# Protective effect of Lai Fu Cheng Qi decoction on severe acute pancreatitis-induced myocardial injury in a rat model

**DOI:** 10.3892/etm.2015.2250

**Published:** 2015-02-03

**Authors:** NAN LI, YING TIAN, CHUNLI WANG, PENG ZHANG, SHENGYI YOU

**Affiliations:** 1 Department of Surgery, General Hospital Affiliated to Tianjin Medical University, Tianjin 300052, P.R. China; 2 Department of General Surgery, The Second Affiliated Hospital of Tianjin University of Traditional Chinese Medicine, Tianjin 300150, P.R. China

**Keywords:** Lai Fu Cheng Qi decoction, severe acute pancreatitis, rats, myocardial protection

## Abstract

The aim of the present study was to evaluate the effects of Lai Fu Cheng Qi decoction on myocardial injury in rats with severe acute pancreatitis (SAP). In total, 30 rats were randomly divided into sham, SAP and decoction treatment groups. SAP was induced by a retrograde pancreatic duct injection of 5% sodium taurocholate in the SAP and decoction treatment groups. Rats in decoction treatment group also received intragastric administration of Lai Fu Cheng Qi decoction. The serum levels of creatine kinase isoenzyme (CK-MB) and lactate dehydrogenase (LDH) were detected using an automatic biochemical analyzer. In addition, myocardial Na^+^-K^+^-ATPase activity was analyzed using a spectrophotometric method and the mitochondrial membrane potential was measured by flow cytometry. Myocardial apoptosis was assessed using a TUNEL assay and pathological changes to the heart and pancreas were detected by hematoxylin and eosin staining. Compared with the rats in the sham group, rats in the SAP and decoction treatment groups exhibited significantly higher levels of serum CK-MB and LDH, apoptosis index and pathological scores, and had significantly lower levels of Na^+^-K^+^-ATPase activity and mitochondrial membrane potential. However, when compared with the SAP group, the serum levels of CK-MB and LDH, the pathological scores of the pancreas and heart, and the myocardial cell apoptosis index in the decoction treatment group were significantly lower. Furthermore, the Na^+^-K^+^-ATPase activity and mitochondrial membrane potential were significantly increased in the decoction treatment group when compared with the SAP group. Therefore, Lai Fu Cheng Qi decoction was shown to exert a protective effect on myocardial injury induced by SAP in rats.

## Introduction

Severe acute pancreatitis (SAP) is a serious surgical disease with a high mortality rate (1). The incidence of SAP has increased markedly in recent years. A number of clinical and experimental studies have been conducted to investigate the pathogenesis of SAP; however, the exact mechanism underlying the pathogenesis of SAP remains unclear (2,3). In addition, the effect of conventional treatments is not satisfactory and there is no specific therapy for SAP (4). Previous studies have shown that a pathological change in pancreatic microcirculation plays an important role in acute pancreatitis, with a local disorder in pancreatic microcirculation, microvascular thrombosis and blood flow blockage able to cause SAP (5,6). Following the occurrence of SAP, leukocytes are activated and accumulate immediately in the pancreatic tissue. Subsequently, inflammatory mediators are released, leading to an increase in pancreatic microvascular spasm and permeability. Thus, pancreatic tissue ischemia-reperfusion damage appears, and through the ‘waterfall cascade’, local acute pancreatitis lesions rapidly develop into systemic inflammatory response syndrome (7). The intestinal tract is the largest reservoir of bacteria, and the intestinal mucosa functions as the barrier for the body to prevent bacteria and endotoxin invasion (8). While the gastrointestinal system is functioning normally, bacteria and endotoxin pass out from the bowels, without bacterial translocation and endotoxin absorption. However, once SAP occurs, the immune barrier is impaired, leading to bacteria and endotoxin invasion into the blood system (9). This further promotes the systemic inflammatory response by activating inflammatory cells and releasing cytokines and inflammatory mediators. A systemic inflammatory response can cause extensive damage to various organs of the body. Previous studies have indicated that pancreatic ischemia may be a key causative factor for hemorrhage and necrosis (10,11). In addition to the necrosis of pancreatic tissues, the microcirculation disorder characterized by ischemia is associated with the development of pancreatic hemorrhage and necrosis (12).

Traditional Chinese medicine has been used for the treatment of acute pancreatitis in China for a number of years. Certain traditional Chinese medicines, with their essential roles in epithelial cell integrity and immune function, have been shown to be important for the maintenance of gut barrier function (13). Lai Fu Cheng Qi decoction is one of the most widely used basic formulas. The decoction is considered to exert certain protective effects on the gastrointestinal tract and systemic organs, with functions including the regulation of gastrointestinal peristalsis, increasing the electroactivity of the intestinal smooth muscle and motilin release (14). Despite wide application, the beneficial effects of this Chinese prescription are controversial and the exact mechanisms underlying the protective function remain unknown. Thus, the aim of the present study was to investigate the protective effect of Lai Fu Cheng Qi decoction on myocardial injury induced by SAP in rats and the possible underlying mechanism.

## Materials and methods

### Reagents and instruments

Lai Fu Cheng Qi decoction was provided by the General Hospital Affiliated to Tianjin Medical University (Tianjin, China). The prescription consisted of 20 g *Rheum palmatum* L., 15 g Mirabilite, 15 g *Magnolia officinalis* Rehd et Wils., 10 g Fructus Aurantii, 15 g Semen Raphani, 10 g Radix Aucklandiae and 10 g *Achyranthes bidentata*. Sodium taurocholate was purchased from Beijing Bioxin Biological Technology Co., Ltd. (Beijing, China). An ATPase detection kit was purchased from Nanjing Jiancheng Biological Engineering Institute (#A070-1; Nanjing, China) and a bicinchoninic acid (BCA) protein assay kit was provided by Beijing Gainingjinnuo Biotechnology Co., Ltd. (Beijing, China). A Tissue Mitochondria Isolation Kit was purchased from the Beyotime Institute of Biotechnology (#C3606; Haimen, China) and a TUNEL *in situ* cell apoptosis detection kit was purchased from Beijing Dingguochangsheng Biotechnology Co., Ltd. (#TUNEL-A1; Beijing, China). Serum biochemical indexes were detected using a Vitros 250 automatic biochemical analyzer (Johnson & Johnson, Rochester, NY, USA). A Cell Lab Quanta SC (CLQSC) Flow Cytometer was obtained from Beckman Coulter (Miami, FL, USA) and an Olympus CK40 inverted microscope was purchased from Olympus Corporation (Tokyo, Japan).

### Animals and grouping

In total, 30 male Sprague-Dawley rats with a body mass of 200–250 g were provided by Beijing Weitong Lihua Experimental Animal Technology Co., Ltd. (Beijing, China). The animals were housed in standard conditions with free access to food and water. All animal experiments were conducted according to the ethical guidelines of Tianjin Medical University, and all efforts were made to minimize animal suffering. The study was approved by the ethics committee of Tianjin Medical University.

The animals were randomly divided into three groups, which included the sham (n=10), SAP (n=10) and the Lai Fu Cheng Qi decoction treatment group (n=10).

### Model preparation and administration

SAP was induced in the SAP and decoction treatment groups by a retrograde pancreatic duct injection of 5% sodium taurocholate (1 ml/kg body weight). Briefly, an incision was made in the abdomen at ~1 cm under the xiphoid midline following anesthetization using 10% chloral hydrate (3 ml/kg; China Pharmaceutical Group Shanghai Reagent Company, Shanghai, China). Once the biliopancreatic duct and the duodenal papilla opening was located, a syringe needle was placed (from the intestinal wall contralaterally to the duodenal nipple) into the biliopancreatic duct through the duodenal papillary opening on the cholo-pancreatic duct. After clipping the cholangitic porta hepatis with a small artery clamp, 5% sodium taurocholate was slowly infused (0.1 ml/min) retrogradely into the pancreatic duct. At 5 min after the injection, the syringe and arterial clamp were removed, the incision was sutured, and the abdomen was closed. In the sham group, an incision was made in the abdomen and the pancreatic tissue was marginally rotated several times prior to closing the abdomen. The abdomen was closed without a sodium taurocholate injection.

At 8 and 16 h after model preparation, the rats in the decoction treatment group received an intragastric administration of Lai Fu Cheng Qi decoction (2.5 ml/kg body weight). As controls, the rats in the sham and SAP groups received an equal volume of saline by intragastric administration.

### Specimen collection

General conditions of the rats following modeling were observed. At 24 h after modeling, three rats in the SAP group and one rat in the decoction treatment group had died. The remaining rats in all three groups were euthanized by intraperitoneal injection of 10% chloral hydrate at 24 h after modeling. The abdomen was opened by surgery, and the pancreas and heart were removed. In addition, blood from the abdominal aorta was collected. The aforementioned procedures were all performed under sterile conditions. The heart tissue was dissected into four sections on ice, and these four sections were used for the detection of Na^+^-K^+^-ATPase activity, mitochondrial membrane potential, myocardial cell apoptosis and pathological examination.

### Detection of serum creatine kinase isoenzyme (CK-MB) and lactate dehydrogenase (LDH) levels

Arterial blood was naturally coagulated at room temperature for 1 h. The blood was subsequently centrifuged at 1006.2 × g for 10 min at 4°C to collect the serum. The CK-MB and LDH concentrations in the serum were detected using the Vitros 250 automatic biochemical analyzer.

### Detection of Na^+^-K^+^-ATPase activity in the myocardial cells

One section of the heart tissue was ground into a homogenate, and the Na^+^-K^+^-ATPase activity was detected using a spectrophotometric method, as previously described (15,16). The experiment was conducted according to the instructions provided in the ATPase detection kit. Briefly, protein levels in the heart homogenate were determined using the BCA protein assay kit. The optical density (OD) of the color developed was measured at a wavelength of 562 nm in a spectrophotometer (Beijing Puxi General Instrument Co., Ltd., Beijing, China). The Na^+^-K^+^-ATPase activity was expressed as U/mgprot and was calculated based on the following formula: Na^+^-K^+^-ATPase activity = [(OD of test tube − OD of control tube)/(OD of standard tube − OD of control tube)] × concentration of standard tube (0.02 μmol/ml) × 6 × 7.8/protein level of the homogenate (mgprot/ml).

### Detection of the mitochondrial membrane potential in the myocardial cells

Using one section of the heart tissue, mitochondria were isolated using the myocardial mitochondria isolation kit and were used to detect the mitochondrial membrane potential. Briefly, the isolated mitochondria were resuspended in 0.5 ml phosphate-buffered saline (Wuhan Boster Biotechnology, Ltd., Wuhan, China), and 10 μl rhodamine 123 working solution (Sigma-Aldrich, St. Louis, MO, USA) was added and incubated with the mitochondria at 37°C in 5% CO_2_ for 15 min. Following incubation, the mitochondrial membrane potential was analyzed on a flow cytometer with an excitation wavelength of 488 nm and an emission wavelength of 633 nm.

### Detection of myocardial cell apoptosis with a TUNEL assay

A TUNEL assay was performed according to the instructions of the apoptosis detection kit. The nuclei of the apoptotic cells exhibited brown/yellow staining, while those of non-apoptotic cells and the negative control were stained blue. The number of apoptotic cells was counted in each group and the apoptotic index (apoptotic cell number/total cell number × 100%) was calculated.

### Hematoxylin and eosin (HE) staining

Pancreatic tissues and one section of the heart tissue were fixed in 4% paraformaldehyde and embedded in paraffin (Beijing Dingguochangsheng Biotechnology Co., Ltd.). The tissues were sliced into sections and stained with HE (Beijing CellChip Biotechnology Co. Ltd., Beijing, China). Briefly, the tissue sections were dewaxed in xylene (Tianjin East Tengen Fine Chemical Reagent Co., Tianjin, China) and rehydrated in graded alcohols. After washing with running water and distilled water, the sections were stained with hematoxylin for 3–5 min. Following a second wash with running water, the sections were differentiated with 1% HCl in 70% alcohol. Subsequently, the sections were stained with eosin for 1–4 min after washing with running water. Following dehydration and differentiation in alcohol, the sections were mounted on cover slips and observed under light microscopy.

### Pathological scoring system

The morphology of the pancreas and heart was evaluated by professional pathologists and the severity of the pathological changes was scored according to previous studies (17,18). According to the pathological changes in edema, infiltration of inflammatory cells, hemorrhage and necrosis in the pancreatic tissues, the following pathological scores were proposed: 0, no pathological changes; 1, mild pathological changes; 2, moderate pathological changes; 3, severe pathological changes; and 4, very severe pathological changes. According to the pathological changes with regard to infiltration of inflammatory cells and myocardial necrosis in the heart tissues, the following pathological scores were proposed: 0, no pathological changes; 1, mild pathological changes; 2, moderate pathological changes; 3, severe pathological changes; 4, very severe pathological changes; and 5, extremely severe pathological changes. The pathological scores of the pancreas (maximum, 16) and heart (maximum, 20) were the total pathological scores, inlcuding the scores for edema, infiltration of inflammatory cells, hemorrhage and necrosis.

### Statistical analysis

Data are expressed as the mean ± standard deviation and SPSS statistical software, version 13.0 (SPSS, Inc., Chicago, IL, USA) was used for statistical analysis. The mortality rates were analyzed using Fisher’s exact probability for a 2×2 contingency table. Comparisons between groups were analyzed using one-way analysis of variance. P<0.05 was considered to indicate a statistically significant difference.

## Results

### General conditions of the rats following modeling

Following the induction of SAP in the rats, the general conditions of the rats were observed. In the sham group, the general conditions of the rats prior to and following surgery were good and did not change significantly. However, the general conditions of the rats in the SAP group were relatively poor. Rats in the SAP group exhibited symptoms of piloerection, laziness, depression, lags in responses and shortness of breath. Three rats had died at 24 h after modeling; thus, the mortality rate was 30% in the SAP group. Following treatment with Lai Fu Cheng Qi decoction, the general conditions of the rats in the decoction treatment group were improved. For example, the symptoms of depression and the lags in responses gradually disappeared. Only one rat had died at 24 h after modeling; thus, the mortality rate was 10% in the decoction treatment group. However, there was no statistically significant difference in the morbidity rate between the SAP and decoction treatment groups (data not shown), indicating that the general conditions of the SAP rats were improved by treatment with Lai Fu Cheng Qi decoction.

### Lai Fu Cheng Qi decoction treatment decreases the serum levels of CK-MB and LDH in SAP rats

To determine the effect of Lai Fu Cheng Qi decoction on the serum levels of CK-MB and LDH, the concentrations of CK-MB and LDH in the serum were measured using an automatic biochemical analyzer (Table I). Compared with the sham group, the serum levels of CK-MB and LDH in the SAP and decoction treatment groups increased significantly (P<0.01). However, the levels of CK-MB and LDH in the decoction treatment group were significantly lower compared with those in the SAP group (P<0.01). These results indicated that SAP increased the levels of CK-MB and LDH in the serum; however, this increase was inhibited by administration of Lai Fu Cheng Qi decoction.

### Lai Fu Cheng Qi decoction treatment increases the Na^+^-K^+^-ATPase activity of myocardial cells in SAP rats

To evaluate the effect of Lai Fu Cheng Qi decoction on Na^+^-K^+^-ATPase activity, the Na^+^-K^+^-ATPase activity of the myocardial cells was detected using a spectrophotometric method. As shown in Table II, the Na^+^-K^+^-ATPase activity of the myocardial cells in the SAP group was significantly lower compared with that of the sham group (P<0.01). In addition, the Na^+^-K^+^-ATPase activity in the decoction treatment group was significantly lower compared with that in the sham group (P<0.01). However, the decoction treatment group had a significantly higher level of Na^+^-K^+^-ATPase activity when compared with the SAP group (P<0.01). These results indicated that SAP reduced the Na^+^-K^+^-ATPase activity of myocardial cells, while Lai Fu Cheng Qi decoction was able to reverse the effect of SAP by increasing the Na^+^-K^+^-ATPase activity.

### Lai Fu Cheng Qi decoction treatment increases the myocardial mitochondrial membrane potential

**Figure 1:**
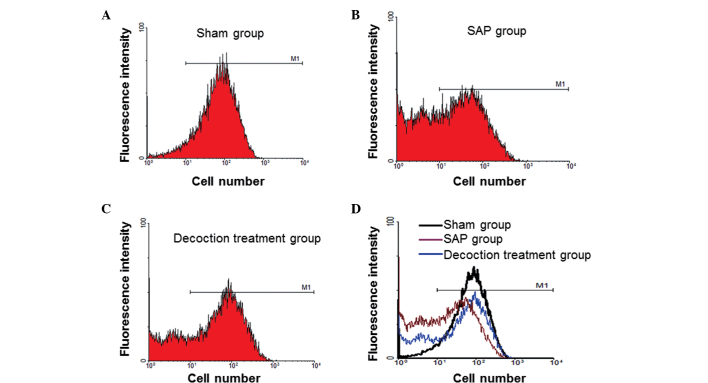
Flow cytometric analysis showing the mitochondrial membrane potential of the myocardial cells. Representative flow cytometry results from the (A) sham, (B) SAP and (C) decoction treatment groups. (D) Comparison among the sham, SAP and decoction treatment groups. SAP, severe acute pancreatitis.

To analyze the effect of Lai Fu Cheng Qi decoction on the mitochondrial membrane potential, mitochondria of the myocardial cells were isolated and the mitochondrial membrane potential was measured by flow cytometric analysis. Representative flow cytometry results are shown in Fig. 1. Cells that did not exhibit a decrease in mitochondrial membrane potential were counted and the percentage was calculated (Table III). In the SAP group, the ratio of cells without a decrease in mitochondrial membrane potential was significantly lower compared with the sham group (P<0.01). Following treatment with Lai Fu Cheng Qi decoction, the ratio of cells without a decrease in mitochondrial membrane potential was significantly higher when compared with the SAP group (P<0.05). Compared with the rats in the sham group, the rats in the decoction treatment group exhibited a significantly lower ratio of cells without a decrease in mitochondrial membrane potential. These results indicated that the mitochondrial membrane potential of myocardial cells decreased following SAP induction, while Lai Fu Cheng Qi decoction was able to reverse the effect of SAP by increasing the mitochondrial membrane potential.

### Lai Fu Cheng Qi decoction treatment decreases the rate of apoptosis of myocardial cells in SAP rats

**Figure 2:**
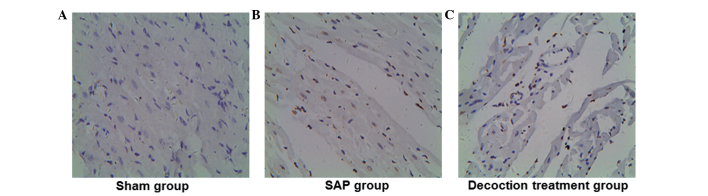
Apoptosis analysis of the myocardial cells in the (A) sham, (B) SAP and (C) decoction treatment groups, as determined using a TUNEL assay (TUNEL staining; magnification, ×400). Representative TUNEL assay results are shown, and cells with brown/yellow staining in the nuclei were determined to be apoptotic cells. SAP, severe acute pancreatitis.

To investigate the effect of Lai Fu Cheng Qi decoction on the rate of myocardial apoptosis in rats with SAP, a TUNEL assay was performed. Representative TUNEL assay results are shown in Fig. 2. The nuclei of the apoptotic cells were stained brown/yellow, while those of non-apoptotic cells and the negative control were stained blue. In the sham group (Fig. 2A), the majority of the nuclei in the myocardial cells were stained blue and only a few myocardial cells exhibited brown/yellow staining in the nuclei. However, in the SAP group, a large number of myocardial cells exhibited brown/yellow staining in the nuclei (Fig. 2B). In addition, the number of cells with brown/yellow staining in the decoction treatment group was higher compared with that in the sham group, whereas the number was lower when compared with the SAP group (Fig. 2C). The number of apoptotic cells was counted in each group and the apoptotic index (apoptotic cell number/total cell number × 100%) was calculated. As shown in Table IV, the apoptosis index in the SAP group was significantly higher when compared with the sham group (P<0.01). Although the apoptosis index of the decoction treatment group was significantly higher compared with that of the sham group (P<0.01), when compared with the SAP group, the apoptosis index of the decoction treatment group decreased significantly (P<0.01). These results indicated that Lai Fu Cheng Qi decoction treatment alleviated the rate of apoptosis induced by SAP in myocardial cells.

### Lai Fu Cheng Qi decoction treatment alleviates the pathological features of the pancreas and heart in SAP rats

**Figure 3:**
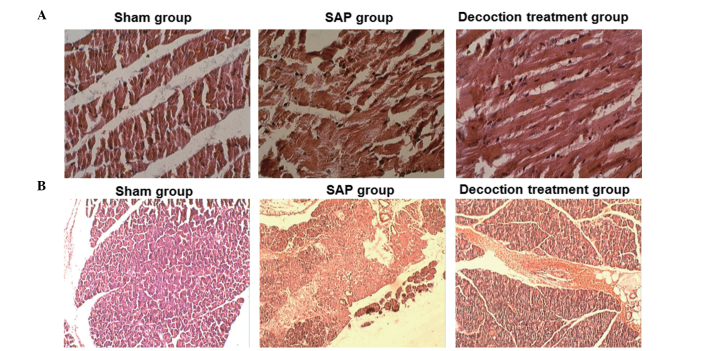
Pathological changes of the pancreas and heart, as determined by hematoxylin and eosin (HE) staining. Representative HE staining results of the (A) pancreatic and (B) heart tissues from the sham, SAP and decoction treatment groups (HE staining; magnification, ×400). SAP, severe acute pancreatitis.

To detect the effects of Lai Fu Cheng Qi decoction on the pathological changes to the pancreas and heart, HE staining was performed. Representative HE staining results are shown in Fig. 3. The morphology of the pancreas and heart was evaluated by professional pathologists. The sham group exhibited a normal morphology of the pancreas (Fig. 3A), and no marked pathological changes were observed. By contrast, the SAP group revealed morphological changes of interstitial edema, interstitial hemorrhage, hemorrhage, necrosis and fat necrosis. The necrotic area was infiltrated with inflammatory cells, which consisted primarily of neutrophils, and the lobular structure was destructed. Following treatment with Lai Fu Cheng Qi decoction, the pathological features of the pancreas in the decoction treatment group were found to improve. Similarly, as shown in Fig. 3B, the sham group exhibited a normal morphology of the myocardial tissue and no significant pathological changes were observed. However, in the SAP group, the myocardial fibers were significantly degenerated and swelled. The majority of the myocardial fiber structures had disappeared and a number of the myocardial membranes were broken. The myocardial fibers were arranged disorderly and the blood vessels exhibited congestion. The aforementioned pathological changes were consistent with the pathological features of myocardial injury induced by SAP. Following treatment with Lai Fu Cheng Qi decoction, the pathological features of the heart in the decoction treatment group were improved. However, edema and mild degeneration of the myocardial fibers was observed in the decoction treatment group, and a number of myocardial fibers had lost cross striations.

In addition, the severity of the pathological changes was scored. The pathological scores of the pancreas and heart are shown in Table V. When compared with the sham group, the pathological scores of the pancreas and heart in the SAP and decoction treatment groups were significantly higher (P<0.01). However, following treatment with Lai Fu Cheng Qi decoction, the pathological scores of the pancreas and the heart in the decoction treatment group were significantly lower compared with those in the SAP group (P<0.01).

Collectively, these results indicated that SAP induced injury in the pancreas and the heart; however, these injuries were alleviated following treatment with Lai Fu Cheng Qi decoction.

## Discussion

Traditional Chinese medicine has been used for the treatment of SAP for a number of years (19). In recent years, the effect of a decoction and the component drugs have been studied systematically, with significant progress achieved. The effect of decoction on SAP is considered to be achieved by promoting gastrointestinal peristalsis, protecting the intestinal barrier function, inhibiting the functions of cytokines and other inflammatory mediators, improving pancreatic microcirculation, protecting pancreatic cells and subcellular organelles and inhibiting the function of pancreatic enzymes (20,21). Lai Fu Cheng Qi decoction exerts specific effects in addition to the aforementioned effects. Lai Fu Cheng Qi decoction can increase the blood flow to the heart, enhance the removal of oxygen free radicals, reduce endotoxin release, inhibit cytokines induced by endotoxin and contribute to inflammation attenuation (22). These effects can further restore gastrointestinal peristalsis, eliminate intestinal toxicant accumulation and reduce endotoxin absorption (7). Additionally, Lai Fu Cheng Qi decoction is known to be effective for the treatment of SAP (23). According to clinical observations, following the application of this decoction, the abdominal symptoms in patients with SAP are relieved through the excretion of stool and the reduction in endotoxin absorption. Furthermore, the possibility of acquiring multiple organ dysfunction syndrome is reduced. In the present study, the levels of serum CK-MB and LDH, the mitochondrial membrane potential, Na^+^-K^+^-ATPase activity, myocardial cell apoptosis and the pathological changes to the pancreas and the heart were examined, in order to further investigate the protective mechanism of Lai Fu Cheng Qi decoction on SAP-induced myocardial injury.

Following the establishment of SAP, the general conditions of the rats were observed. In total, three rats from the SAP group and one rat from the decoction treatment group had died at 24 h after modeling. However, there were no statistically significant differences in the morbidity rate between the SAP and decoction treatment groups, which may be partly due to the small sample size included in the study. In addition, the treatment time of decoction in the current study design was only 24 h, which was relatively limited. Therefore, further studies with a larger sample size and a longer treatment time period are required.

Levels of CK-MB and LDH in the serum can be used as markers of myocardial injury (24,25). In the present study, changes in the serum levels of CK-MB and LDH were also detected. The results demonstrated that the serum levels of CK-MB and LDH in the SAP rats treated with Lai Fu Cheng Qi decoction were significantly decreased. This result indicated that the upregulation of serum CK-MB and LDH levels in SAP rats with myocardial injury was inhibited by Lai Fu Cheng Qi decoction treatment. Therefore, the results provide preliminary experimental evidence that Lai Fu Cheng Qi decoction exerts myocardial protective effects in rats with SAP.

A decrease in the mitochondrial membrane potential is a sign of early apoptotic events (26). Rhodamine 123 is an indicator of mitochondrial transmembrane potential. Through the fluorescence signal intensity, the changes in mitochondrial membrane potential and cell apoptosis at an early stage can be detected. In the present study, the percentage of myocardial cells that did not undergo a decrease in the mitochondrial membrane potential in the decoction treatment group was significantly higher compared with that of the SAP group. These results indicated that Lai Fu Cheng Qi decoction was able to inhibit the decrease in mitochondrial membrane potential induced by SAP, subsequently inhibiting the apoptosis of myocardial cells.

Na^+^-K^+^-ATPase is an antiporter enzyme located in the plasma membrane of all animal cells. The Na^+^-K^+^-ATPase aids the maintenance of a resting potential and regulates the cellular volume (27). The enzyme also functions as a signal transducer/integrator to regulate the mitogen-activated protein kinase signaling pathway, reactive oxygen species and intracellular calcium. In the present study, the myocardial cell Na^+^-K^+^-ATPase activity in the Lai Fu Cheng Qi decoction treatment group was higher when compared with the SAP group that did not receive treatment. Therefore, Lai Fu Cheng Qi decoction may improve the activity of the Na^+^-K^+^-ATPase, prevent intracellular calcium overload, sustain the matrix volume stability and inhibit ischemia and hypoxia; thus, exerting myocardial protective effects. The myocardial protective effects of Lai Fu Cheng Qi decoction were further verified by a TUNEL assay. The results revealed that the apoptosis index of the decoction treatment group decreased significantly when compared with the SAP group.

The pathological changes observed in the pancreatic and myocardial tissues of the rats in the SAP group were consistent with the injuries induced by SAP. Following treatment with Lai Fu Cheng Qi decoction, the pathological features of the pancreas and the heart were alleviated. However, the features of edema and neutrophil infiltration remained in the decoction treatment group. The severity of the pathological changes was graded. Consistently, the pathological scores of the pancreas and heart in the decoction treatment group were significantly lower compared with those in the SAP group, indicating that Lai Fu Cheng Qi decoction was able to improve the pathological changes induced by SAP.

In conclusion, the present study demonstrated that Lai Fu Cheng Qi decoction is able to effectively decrease the serum levels of CK-MB and LDH, increase Na^+^-K^+^-ATPase activity, inhibit a decrease in mitochondrial membrane potential and inhibit the apoptosis of myocardial cells, subsequently reducing the myocardial injuries induced by SAP. The present study is a pilot study that included a limited number of rat models. Future studies with a larger sample size and prospective trials are required to further investigate the mechanisms underlying the protective effects of Lai Fu Cheng Qi decoction on SAP-induced myocardial injury.

## Figures and Tables

**Table I:** Effect of Lai Fu Cheng Qi decoction on the serum CK-MB and LDH levels.

Group	Cases (n)	CK-MB (μ/ml)	LDH (μ/l)
Sham group	10	639.33±37.72	593.17±45.43
SAP group	7	2133.29±111.60^a^	1873.71±141.86^a^
Decoction treatment group	9	1332.78±120.39^a^,^b^	953.11±74.23^a^,^b^

Results are expressed as the mean ± standard deviation.

a P<0.01, vs. sham group;

b P<0.01, vs. SAP group.

CK-MB, creatine kinase isoenzyme; LDH, lactate dehydrogenase; SAP, severe acute pancreatitis.

**Table II:** Effect of Lai Fu Cheng Qi decoction on Na^+^-K^+^-ATPase activity.

Group	Cases (n)	Na^+^-K^+^-ATPase activity (U/mgprot)
Sham group	10	10.65±0.96
SAP group	7	4.62±0.51^a^
Decoction treatment group	9	8.06±0.73^a^,^b^

Results are expressed as the mean ± standard deviation.

a P<0.01, vs. sham group;

b P<0.01, vs. SAP group.

SAP, severe acute pancreatitis.

**Table III:** Effect of Lai Fu Cheng Qi decoction on the mitochondrial membrane potential.

Group	Cases (n)	Cells without a decrease in mitochondrial membrane potential (%)
Sham group	10	95.13±1.94
SAP group	7	45.59±2.97^a^
Decoction treatment group	9	66.61±3.99^a^,^b^

Results are expressed as the mean ± standard deviation.

a P<0.01, vs. sham group;

b P<0.01, vs. SAP group.

SAP, severe acute pancreatitis.

**Table IV:** Effect of Lai Fu Cheng Qi decoction on the apoptosis of myocardial cells.

Group	Cases (n)	Apoptosis index (%)
Sham group	10	1.76±0.54
SAP group	7	31.62±4.68^a^
Decoction treatment group	9	24.06±3.75^a^,^b^

Results are expressed as the mean ± standard deviation.

a P<0.01, vs. sham group;

b P<0.01, vs. SAP group.

SAP, severe acute pancreatitis.

**Table V:** Effect of Lai Fu Cheng Qi decoction on the pathological scores of the pancreas and heart.

Group	Cases (n)	Pathological scores of the pancreas	Pathological scores of the heart
Sham group	10	1.67±0.82	0
SAP group	7	12.14±1.35^a^	2.86±0.69^a^
Decoction treatment group	9	6.33±1.41^a^,^b^	1.67±0.50^a^,^b^

Results are expressed as the mean ± standard deviation.

a P<0.01, vs. sham group;

b P<0.01, vs. SAP group.

SAP, severe acute pancreatitis.
